# Examining the influence of information overload on consumers’ purchase in live streaming: A heuristic-systematic model perspective

**DOI:** 10.1371/journal.pone.0284466

**Published:** 2023-08-04

**Authors:** Guihua Zhang, Junwei Cao, Dong Liu

**Affiliations:** 1 Department of Business, Yeungnam University, Gyeongsan, Korea; 2 School of Business, Yangzhou University, Yangzhou, China; 3 Department of Global Business, Yeungnam University, Gyeongsan, Korea; Jiangsu University, CHINA

## Abstract

In recent years, the number of live streaming platforms and the number of viewers have exploded. For example, in China, there are already more than 100 live streaming platforms as well as more than 300 million consumers. In live commerce, streamers use ‘information overload’ to affect purchase decisions, by making it difficult for consumers to think systematically; however, the mechanism of impact in this way has not been confirmed. In order to clarify how information overload affects users’ purchasing decisions in live commerce, this study introduces information overload from the perspective of "Heuristic-systematic model" to develop a research model. And 400 respondents were randomly recruited through various SNS platforms in Guangdong Province, China from July 1 to July 20, 2022 using a random survey method, and finally 297 valid sample data were obtained. To verify the validity of the model and to illustrate the impact of information overload marketing on consumer purchase decisions in live commerce this study conducted an empirical analysis using Smart PLS 3.0. The findings show that perceived product quality and fit, and streamer influence and expertise, positively affect consumers’ purchase intentions. The information overload strategy can significantly reduce consumers’ perceptions of merchandise fit and enhance their perceptions of streamer influence and expertise. The results of this study provide a theoretical basis for marketing strategies in live commerce, and enrich literature in the field of marketing.

## 1. Introduction

Live commerce is a model of social commerce wherein products are sold through live webcasts [1]. It uses the high interactivity of social media to engage with consumers in real-time, leading to swift transactions [2]. In recent years, the number of live streaming platforms and viewers have increased. For example, in China, there are already more than 100 live streaming platforms and more than 300 million consumers, with the market having grown from $3 billion in 2016 to $15 billion in 2020 [3]. At the same time, a number of web streamers with great commercial value have emerged, whose broadcasts can attract tens of millions of consumers, with the turnover exceeding even ten million dollars [4].

Currently, marketing in live commerce is dominated by two directions: utilitarian value and emotional value [5, 6]. Utilitarian value is an important antecedent to predicting user purchase intent [7]. Current webcasting technology can help streamers present detailed and vivid product information, Such a working environment and job satisfaction also increase the enthusiasm of streamer [8], and reduce consumers’ doubts about the utilitarian value of products, and increase their willingness to purchase [2, 6, 7]. The emotional value provided to consumers by online streamers in live commerce positively predicts users’ purchase intentions [5]. Live broadcast platforms focus on building virtual emotions and interactions between consumers and streamers by increasing the attractiveness of the streamer, improving the enjoyment feeling, constantly stimulating consumers, triggering their emotional commitment and stream experience, establishing their identity to the streamer, and prompting them to generate emotional commitment to the streamer, and eventually purchase [9]. An empirical study showed that Korean consumers’ emotional identification with streamers increases their purchase intention [9].

During a live broadcast, the streamer creates a stimulating atmosphere from utilitarian and emotional values, repeatedly emphasizing the excellent performance and price advantage of the product, reminding consumers of the limited time, and constantly encouraging them to make a purchase decision [2]. At the same time, the streamer will also repeatedly use intimate titles to address consumers (such as family or dear) to establish a close virtual relationship, so that consumers do not see the streamer as the provider of the product, but as a family member or friend they can trust [10]. Such a marketing model may put consumers in a state of information overload. Information overload has been shown to confuse consumers in their decision making [11], whereby they may purchase products which they do not require immediately.

However, the impact of information overload on consumers’ purchase decisions remains controversial. A study was conducted on consumers by building eight different shopping sites; the results indicated that more product information increases consumer trust, thus reducing consumer price perception, and promoting purchase [12, 13]. Another study found that information overload in mobile web shopping reduces consumer trust and purchase intention [14]. Yet, the Heuristic-systematic model has not been used to explain the relationship between information overload and users’ purchasing decisions. Therefore, the adoption of a new research perspective for validation in a new online shopping environment can help further explain the mechanisms by which information overload affects consumers’ purchase decisions. Therefore, this study poses the following research question:

RQ1: How does information overload affect purchasing decisions in live commerce?

To address this research question, we draw on the heuristic-systematic model (HSM) of information processing. HSM theory explains the differences between heuristic and systematic information processing and why one systematically examines messages while heuristically processing their surface cues [12]. By distinguishing between product features as systematically processed information and anchor influence as heuristically processed information, HSM provides an appropriate theoretical framework to illustrate the consumer decision process in live web sales. In addition, HSM is based on the premise that "people rarely process information under perfect conditions and there are both environmental and cognitive constraints to information processing" [12], and that information overload may lead to consumer confusion, so it is appropriate to introduce information overload into the HSM construct in this study.

This study provides empirical evidence that consumer perceived product quality and perceived fit can act to reflect systematic information processing cues, and that anchor influence and expertise can act to reflect heuristic information processing cues. The potential contributions of this study are as follows: 1) To further clarify the impact of information overload on users’ purchase decisions in live commerce. 2) To confirm the mechanism of information overload marketing on consumer purchases in live commerce, enrich the literature on marketing research, and provide a theoretical basis and practical guidance for live commerce practitioners to develop marketing plans.

## 2. Literature review and hypothesis development

### 2.1. Heuristic-systematic model

The heuristic-systematic model (HSM) is a dual-process theory used to analyze the formation of individual judgments with limited cognitive resources [15, 16]. HSM suggests that changes in individuals’ attitudes when confronted with persuasive messages are a joint result of heuristic and systematic modes of information processing [17]. Heuristic information processing means that "people consider a number of information cues and form judgments based on these cues" [18], meaning that when heuristic information processing is used, people make judgments based on simple cues (e.g., characteristics of the information source) [15]. That is, because of limited cognitive resources, people choose to spend less cognitive effort to make quick judgments through simple rules and experiences in order to minimize information processing [18]. Systematic information processing indicates that information receivers require significant cognitive effort to assess the validity of information to make judgments [18]. This means that when people have sufficient motivation, ability, and cognitive resources, they tend to engage in systematic information processing [19].

Recently, HSM has been used widely in online marketing studies. Several studies have analyzed the impact of online reviews on users’ purchase decisions based on HSM [20–22]. It is generally noted in these studies that judgments about the nature of the information itself, are commonly found in users’ systematic information processing, while consideration of the characteristics of the source (e.g., credibility of the source, objectivity of comments, etc.), is part of users’ enlightened information processing [21, 22]. In addition, both heuristic and systematic information processing increase consumers’ purchase decisions, but the latter has a stronger effect than the former [21]. Another study used a heuristic-systematic model to study the success factors of YouTube weblebrity marketing, which indicated that source credibility cues (e.g. weblebrity expertise, weblebrity credibility, level of interaction, etc.) appeared in users’ heuristic information processing of the product, while perceived product quality and product knowledge appeared in users’ systematic information processing of the product [23]. However, the antecedents influencing which information processing methods users have adopted in past studies are unclear, and clarifying this point would help in the development of the theory.

### 2.2. Information overload in marketing

Information overload first appeared in Gross’s book [24]. With the rapid development of information technology, information overload is becoming increasingly common in daily life [25]. Information overload occurs when the amount of information that needs to be processed in a given period of time is greater than the individual’s information processing capacity [26]. It occurs when individuals retrieve and analyze information, and subsequently make decisions. The quality, complexity, and ambiguity of information within a certain period of time, as well as an individual’s knowledge and experience reserves, are closely related to the generation of information overload [26, 27]. Information overload can cause individuals to suffer from fatigue, anxiety, fear of missing out, and other psychological problems, leading to poor decisions [25, 28] and even to distrust of the web [29].

Numerous studies have confirmed that information overload has a significant impact on consumer decisions. The quality of people’s decisions is positively related to the amount of information they receive within a certain range. When information overload occurs, the quality of decision making decreases significantly [30]. The effect of information overload on consumer behavior in different environments is controversial [13]. Information overload can reduce consumer attention and affect shopping decisions [11]. Conversely, when exploited by marketers, information overload can positively influence consumers’ purchase intentions [12–14]. In one study, eight different shopping sites were tested on consumers, and the results indicated that more product information increased consumer trust, which reduced price awareness, and led to purchases [12, 13]. Another study based on 1,396 people pointed out that information overload can directly increase consumers’ willingness to purchase but can also indirectly reduce it by increasing consumers’ perceived risk [13]. In an online shopping environment, information overload may also be related to consumers’ purchase intention in an inverted U. Low-intensity information overload does not significantly trigger consumers’ purchase intention, whereas medium-intensity information overload is effective in developing trust and purchase intention [14]. Therefore, in this study, we use information overload as a marketing approach to elucidate its impact on consumers’ shopping intentions in the context of live commerce from an HSM perspective.

### 2.3. Research model and hypothesis development

**Fig 1:**
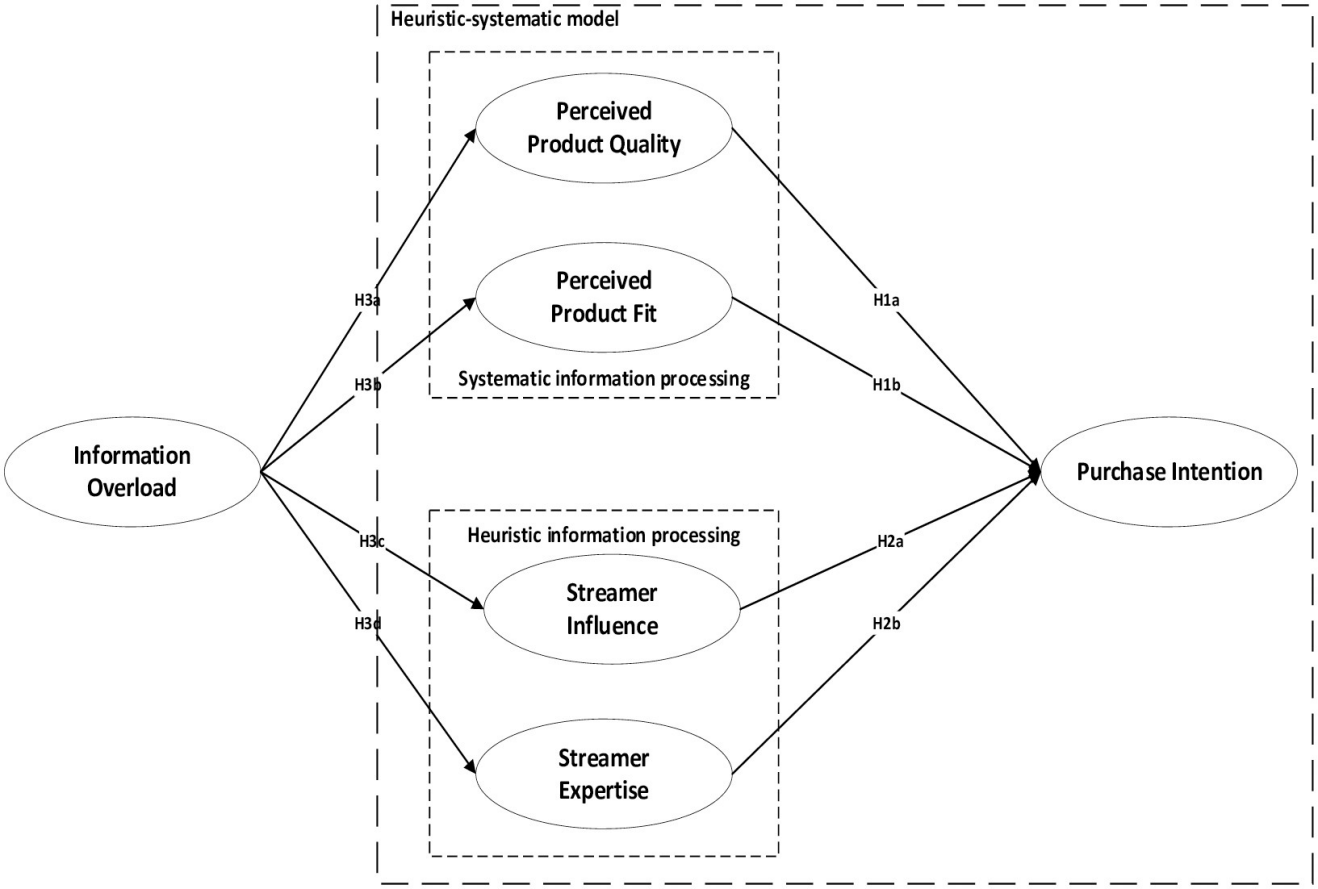
The proposed research model.

This study uses HSM as the theoretical basis, combining it with the relevant literature and research background, to develop a research model to demonstrate the impact of information overload marketing on consumer purchase intention in live commerce (see Fig 1). In a live-commerce environment, consumers systematically think about the information related to the product itself to make purchase decisions, and heuristically make purchase decisions based on the personal characteristics of the streamer. Streamers can influence users’ information-processing patterns through information overload marketing.

#### 2.3.1. Systematic information processing

One study that investigated the adoption of preventive behaviors by people with Covid-19, confirmed that information overload influences behavioral intentions by reducing systematic information processing [15]. During systematic information processing, people think about the arguments themselves; for example, the better argued the core information is, the more it influences the information receiver’s decision making [18]. Users use live commerce to gain utilitarian value through shopping, so the core features of the product are an important factor for consumers to consider [31, 32].

Some scholars in the online shopping environment have found that perceived quality is closely related to trust and purchase intention [33]. People’s perception of product quality directly affects their emotions, satisfaction and perceived value, and ultimately has a great impact on their purchasing decisions [34, 35]. In live commerce, products need to be competitive not only in price but also in quality, for customers to perceive their value and their suitability to consumer needs, prompting positive purchase decisions [36]. Therefore, the following hypothesis was proposed in this study:

H1a: Consumer perception of product quality has a positive impact on purchase intention.

Perceived product fit is defined as the extent to which consumers are unable to assess whether the product attributes match their preferences, and their preferences make them more likely to desire certain products or product attributes [37]. Many consumers are unsure of the fit between their preferences and the functional characteristics of the product, but advanced information technology enables consumers to investigate their potential fit with the product before purchase. If consumers find that the product does not match their preferences, they will not purchase it, so providing product match information becomes an important strategic decision for sellers [38]. Sellers need to make efforts to reduce consumers’ concerns about product incompatibility when selling products [39], and consumers are more likely to make purchase decisions when they perceive that the product is appropriate for them. Therefore, we propose the following hypothesis:

H1b: Consumer perception of product fit has a positive impact on purchase intention.

#### 2.3.2. Heuristic information processing

A user’s heuristic information processing process is expressed as a reflection of the characteristics of the information source [21, 22]. Online sellers’ communication skills, influence, authenticity, and expertise influence consumer attitudes [40].

The seller’s influence is an important factor in guiding consumers’ purchases. Influential celebrities have proven valuable on the Internet and social media, and shopping guidance from these people is more likely to trigger consumers’ willingness to purchase [41]. When influential sellers use a product, followers are more likely to feel the value of using that product and are more inclined to purchase it [42]. Research has shown that attitude, emotion and reinforcement are important factors in enhancing user use [43]. In live commerce, reviews of products by influential streamers can quickly encourage consumers to trust the product and make a purchase [40, 44]. When it is difficult for consumers to judge by themselves, they rely on the recommendations of streamers, and the more influential the streamers are, the more they are likely to be trusted by consumers. Therefore, we propose the following hypothesis:

H2a: The influence of the streamer positively affects the consumer’s purchase intention.

The expertise of the salesperson is an important factor in influencing a user’s intention to purchase a product [40]. The expertise of online celebrities is considered important for product sales success, so they often present themselves as experts in areas such as "gaming,” "fitness,” "makeup" or "fashion," and regularly share product information with their online followers through video blogging, so that their followers are more likely to trust them and feel the value of using the product [45]; with raised expectations, they will be more inclined to purchase [46, 47]. Therefore, we propose the following hypothesis:

H2b: Streamers’ expertise positively influences consumers’ purchase intentions.

#### 2.3.3. Information overload

Information overload negatively impacts the systematic information processing of individuals [15]. When purchasing goods, consumers need to systematically and comprehensively evaluate various types of information, such as product quality and product fit. However, in commodity information processing, one is exposed to a lot of information, and careful attention, deep thinking, and deep reasoning are highly dependent on individual abilities, often requiring more energy to process the information [17, 31]. In live commerce, when consumers evaluate product information, streamers often repeatedly hype the high value of the product in a short period of time, shaping an intimate environment and reminding users of limited time, so that they are unable to think, and rush to purchase [48]. This can easily lead to user information overload because the amount of information exceeds the user’s information processing capacity, which can lead to user fatigue and cognitive dissonance [25], thereby making consumers’ attention drop [11]. Therefore, under the effect of information overload, consumers’ ability to perceive product value and fit may be reduced. Therefore, we propose the following hypothesis:

H3a: Information overload negatively affects consumers’ perception of product quality.
H3b: Information overload negatively affects consumers’ perception of product fit.

Processing information through source cues is a heuristic information processing process. It has been shown that information overload has a positive effect on an individual’s heuristic information processing process [15]. Consumers with information overload are fatigued, and refuse to expend energy to evaluate products systematically. At this point consumers can only rely on simple information to make purchase decisions. In live commerce, streamers are the core source of commodity information, and one of their key characteristics is simple information that can be identified without much effort [32]. For example, the influence of a seller can be judged quite simply and intuitively from the number of followers and activity participation [40]. Therefore, we propose the following hypothesis:

H3c: Information overload positively affects consumer perceptions of the streamer’s influence.
H3d: Information overload positively affects consumer perceptions of the streamer’s expertise.

## 3. Research methods

### 3.1. Sampling and data collection

In the context of this study, a questionnaire was prepared, based on previously validated scales. 1) The questions were administered on a 5-point Likert scale. 2) As the source language of the questions was English, we asked two linguists, proficient in the language, to translate the questionnaire from Chinese to English to ensure that the Chinese expressions were error-free and easy to understand. 3) We set reverse questions in the questionnaire to detect invalid questionnaires. 4) We randomized the questions to avoid errors caused by random responses and psychological implications as much as possible. 5) Experts from relevant fields were invited to review the questionnaire. The resulting questionnaire is presented in Supporting Information files.

According to the data of the Baidu index (http://index.baidu.com), we found that between April-June 2022, internet users in Guangdong Province, China, paid the most attention to live-streaming of goods. Therefore, this study identifies consumers living in Guangdong Province, China, who use live-streaming commerce, as the target survey respondents.

We consulted the ethical rules of the university ethics review committee regarding the conduct of questionnaires and followed these rules to design a survey process that would be exempt from ethical review: 1) This experiment was completely anonymous; 2) participants were informed of the purpose of this study before being surveyed; 3) only necessary data were collected for this study, and kept strictly confidential; 4) material rewards were given to respondents who participated; 5) Data are only available to researchers who meet the requirements.

We conducted the survey directly from July 1 to July 20, 2022, after completing the preparatory work. We used a random survey method, in which 400 respondents were randomly recruited through various SNS platforms. We finally received a total of 378 questionnaires; excluding those with no live shopping experience, no purchase experience persuaded by the streamer, repeated answers, biased reverse questions, and less than 2 minutes of answering time, yielded a total of 297 valid questionnaires (78.5%).

### 3.2. Data analytical tool

First, we conducted a descriptive analysis of the demographic characteristics of the sample. Second, indicators related to the quality of the model were assessed. Finally, the proposed hypotheses were tested.

There are two types of structural equation models, the covariance-based structural equation model (CB-SEM) and the variance-based partial least squares structural equation modeling (VB-SEM). In this study, variance-based PLS-SEM and the corresponding software package SmartPls 3.0 were used for data analysis. The main reasons for this are: 1) PLS-SEM is more suitable than CB-SEM for measuring structural equation models with more than 6 variables [49]. 2) PLS-SEM is more suitable for a wider range of data characteristics than CB-SEM, especially for handling non-normally distributed data [49]. 3) PLS-SEM is more suitable for small-sample measurements. 4) PLS-SEM is more suitable for exploratory studies in the early stages of theory construction [49].

In this study, six variables were used in the proposed model. Second, a multivariate normality analysis was performed on the data collected in this study, using a web calculator to measure the distribution of the data (https://webpower.psychstat.org/); the results show Mardia’s multivariate skewness (β = 24.980, p <0.05), and multivariate kurtosis (β = 350.420, p <0.05), which suggests multivariate non-normality [50]. Additionally, this is an exploratory study. In summary, this study is applicable to data analysis using PLS-SEM.

### 3.3. Common method bias test

As shown in Table 1, a total of 297 valid questionnaires was collected for conducting the experiment. Among them, 149 (50.2%) were male and 148 (49.8%) were female; 58 (19.5%) were aged 20–29, 79 (26.6%) were aged 30–39, and 93 (31.3%) were aged 40–49; 96 (32.3%) had specialist degrees, and 119 (40.1%) had Bachelor’s degrees. The largest number of people, i.e., 132 (44.4%), had incomes below RMB 3999, followed by those with incomes between RMB 4000–8000, i.e., 110 (37%).

**Table 1:** Demographic details of the survey respondents.

Items	Options	Frequency (Total = 297)	Percentage (%)
**Gender**	Male	149	50.2
	Female	148	49.8
**Age**	20–29	58	19.5
	30–39	79	26.6
	40–49	93	31.3
	50 years or above	67	22.6
**Education**	Specialist degrees	96	32.3
	Bachelor’s Degree	119	40.1
	Master or Phd Degree	82	27.6
**Income (Per month)**	RMB 1000–2000	67	22.6
	RMB 2000–4000	65	21.9
	RMB 4000–6000	57	19.2
	RMB 6000–8000	53	17.8
	More than RMB 8000	55	18.5

We conducted a paired t-test to detect nonresponse bias in the demographic data of the first and last 25 respondents, and the results showed no significant difference; therefore, nonresponse was not a serious problem in this study.

Common method bias is a common problem in questionnaires, and two methods were used to measure it in this study. As per Harman’s single factor analysis [51], the percentage of variables extracted was 21.9% (less than 40%). We also used FLL-VIF to measure the common method bias in PLS-SEM [50, 52], and all the VIF values were below 3.3. The results of both testing methods indicated that common method bias was not a serious problem.

## 4. Results analysis

### 4.1. Measurement model

We evaluated composite reliability (CR), average variance extracted (AVE), discriminant validity, and outer loading to test the measurement model. As shown in Table 2, the variables of composite reliability > 0.7, Cronbach’s alpha > 0.7, indicating that the internal consistency of the data in this study is qualified. The AVE of the variables > 0.5, outloadings > 0.7, indicating that the convergent validity of the data in this study is qualified [49]. As shown in Table 3, the Fornell and Larcker’s Test and Heterotrait-Monotrait ratio Test (HTMT) were used to measure the discriminant validity of this study. The HTMT values between variables were below the 0.85 threshold, and the square root of each variable AVE was greater than the correlation between its variables [49]. The results of all the analyses were combined, and the study had good overall reliability, convergent validity, and discriminant validity.

**Table 2:** Reliability and validity coefficients for constructs.

Latent variable	Item	Loading	Mean (SD)	Cronbach’s a	CR	AVE	R^2^
IFO	IFO1	0.856	3.03 (1.087)	0.836	0.901	0.753	-
	IFO2	0.916					
	IFO3	0.829					
PPQ	PPQ1	0.859	2.82 (1.111)	0.843	0.905	0.761	0
	PPQ2	0.908					
	PPQ3	0.849					
PPF	PPF1	0.800	3.62 (0.810)	0.801	0.882	0.714	0.130
	PPF2	0.891					
	PPF3	0.842					
SIF	SIF1	0.912	2.77 (0.975)	0.848	0.905	0.761	0.100
	SIF2	0.881					
	SIF3	0.821					
STE	STE1	0.881	2.98 (0.943)	0.838	0.900	0.752	0.122
	STE2	0.915					
	STE3	0.801					
PIT	PIT1	0.835	3.27 (0.754)	0.788	0.872	0.694	0.250
	PIT2	0.895					
	PIT3	0.765					

Abbreviations: IFO-Information overload; PPQ-Perceived Product Quality; PPF-Perceived Product Fit; SIF-Streamer Influence; STE-Streamer Expertise; PIT-Purchase Intention

**Table 3:** Discriminant validity.

Fornell-Larcker Criterion						
	IFO	PPQ	PPF	SIF	STE	PIT
IFO	0.868					
PPQ	0.009	0.872				
PPF	-0.361	0.001	0.845			
SIF	0.317	-0.006	-0.164	0.872		
STE	0.349	-0.035	-0.151	0.098	0.867	
PIT	0.091	0.387	0.255	0.223	0.236	0.833
Heterotrait-Monotrait Ratio						
	IFO	PPQ	PPF	SIF	STE	PIT
IFO						
PPQ	0.039					
PPF	0.42	0.029				
SIF	0.347	0.018	0.183			
STE	0.394	0.076	0.173	0.096		
PIT	0.103	0.45	0.335	0.243	0.263	

Abbreviations: IFO-Information overload; PPQ-Perceived Product Quality; PPF-Perceived Product Fit; SIF-Streamer Influence; STE-Streamer Expertise; PIT-Purchase Intention

### 4.2. Structural model

We first tested for covariance; the VIFs of the variables were all below 3, so covariance was not a major issue in this study. After establishing the reliability and validity of the model and ensuring that covariance was not an issue, this study measured the structural model to test the proposed hypotheses. The results of testing the path coefficients and significant cases of the structural model are presented in Table 4. Perceived product value (β = 0.396, p < 0.001), perceived product fit (β = 0.343, p < 0.001), streamer influence (β = 0.252, p < 0.001), and streamer expertise (β = 0.277, p < 0.001) all significantly and positively influenced users’ purchase intention, and H1a, H1b, H2a, and H2b were all supported. There was no significant effect of information overload on perceived product value (β = 0.009, n.s.), and H3a was not established. Information overload has a significant negative effect on perceived product fit (β = -0.361, p < 0.001), and H3b is supported. Information overload can significantly and positively influence perceived streamer influence (β = 0.317, p < 0.001), supporting H3c. Information overload also had a significant positive effect on perceived streamer expertise (β = 0.349, p < 0.001), supporting H3c. Additionally, none of the control variables had a significant effect on users’ purchase intentions.

**Table 4:** Assessment of the structural model.

Hypothesis	β	STDEV	T Statistics	P Values	Result
H1a: PPQ -> PIT	0.396	0.042	9.468	0.000	Support
H1b: PPF -> PIT	0.343	0.048	7.123	0.000	Support
H2a: SIF -> PIT	0.252	0.047	5.333	0.000	Support
H2b: STE -> PIT	0.277	0.049	5.671	0.000	Support
H3a: IFO -> PPQ	0.009	0.059	0.151	0.880	Reject
H3b: IFO -> PPF	-0.361	0.066	5.458	0.000	Support
H3c: IFO -> SIF	0.317	0.052	6.12	0.000	Support
H3d: IFO -> STE	0.349	0.052	6.752	0.000	Support
Edu -> PIT	-0.035	0.048	0.728	0.467	-
Gender -> PIT	-0.063	0.097	0.654	0.513	-
Income -> PIT	0.026	0.048	0.555	0.579	-
Age -> PIT	-0.014	0.047	0.294	0.769	-

Abbreviations: IFO-Information overload; PPQ-Perceived Product Quality; PPF-Perceived Product Fit; SIF-Streamer Influence; STE-Streamer Expertise; PIT-Purchase Intention

Finally, using Stone-Geisser Q2 [53, 54], predictive relevance was evaluated and the results are presented in Table 5. The Q2_predict value is greater than 0 for Perceived Product Fit (0.117), Streamer Expertise (0.114), and Streamer Influence (0.094), demonstrating that the model is predictively valid [55]. And we tested the goodness of fit (GOF) of the model and used standardized root mean square residuals (SRMR) to check the goodness of fit of the model. The SRMR value of the model in this study is 0.078, which meets the requirement of less than the threshold value of 0.08, and it can be concluded that the fit of this study is acceptable [56].

**Table 5:** PLS predict test results.

	Q²predict	RMSE	MAE
PPQ	**-0.006**	1.01	0.879
PPF	**0.117**	0.947	0.769
SIF	**0.094**	0.958	0.77
STE	**0.114**	0.947	0.774
PIT	**0.002**	1.005	0.846

Abbreviations: IFO-Information overload; PPQ-Perceived Product Quality; PPF-Perceived Product Fit; SIF-Streamer Influence; STE-Streamer Expertise; PIT-Purchase Intention

## 5. Discussion and conclusion

### 5.1. Key findings

This study explores the purchase decision process of live commerce consumers from the perspective of HSM. First, the core features of a product are the factors that promote its purchase, and the consumer systematically evaluates this information to decide whether or not to buy that product. Consumers will measure the quality of goods through technical and performance characteristics when shopping; excellent product quality can increase consumer trust and promote the purchase [57, 58]. Consumers also tend to purchase goods that suit their preferences, therefore product fit exerts a significant influence on the willingness to purchase [10].

Second, in addition to the core characteristics of the goods, consumers heuristically evaluate the characteristics of the transmitters of the goods’ characteristics (e.g., streamers) to make purchase decisions. The influence of a streamer can be judged, based on the number of followers and the level of participation in the event [40], which can increase consumers’ trust in products in a short period of time, ultimately influencing their purchasing decisions [40, 44]. A salesperson’s expertise can raise the user’s expectations of the product and generate willingness to purchase [47]. If streamers make consumers feel that they have professional knowledge of the product, more consumers are inclined to accept their marketing.

This study aims to explain how information overload marketing by streamers in live commerce affects consumers’ purchase decisions. First, this study found that information overload can significantly reduce users’ perceptions of product fit. With information overload, users become fatigued and cognitively dissonant [25], thereby lacking the energy to process too much information, and making their purchase decisions in a state of confusion [11]. Product fit is the characteristic information of the product, which is the core factor that users need to consider when purchasing products [31, 32], and requires effort to process systematically. Previous studies have confirmed that information overload negatively impacts users’ systematic information processing [15]. Therefore, if the streamer repeatedly hypes the high value of the product within short time intervals, shapes the intimate environment, and reminds the user of the limited time, the user may ignore the product fit and rush to purchase it [48].

Second, this study found that information overload significantly increases users’ perceptions of streamer influence and expertise. Consumers with information overload do not have the energy to process information due to fatigue and may even avoid new information [59]; they avoid systematic evaluation of complex information, preferring to evaluate it through simple clues without expending too much effort [15]. The large amount of product information conveyed in a short period of time, as well as the atmosphere created by a streamer in the live broadcast, will improve consumers’ perception of his/her characteristics. Although this perception may be incorrect in many cases, the number of followers and the level of participation in current activities can easily influence consumer perception of the streamer’s influence [40]. Moreover, the large amount of information and knowledge repeatedly delivered to users in a short period, can also influence their perception of the streamer’s expertise. Combining the above two points, it can be found that information overload marketing seems to be a good choice. When people are overloaded with information, less attention will be devoted to decision making, so it is beneficial for the platform and the streamer to increase sales in a short time.

Finally the results of this study did not confirm the significant effect of information overload on users’ perceived product quality. A possible explanation is the positive correlation between knowledge base and information overload [26], though the perception of product quality is the core information of purchase, and is based on long-term experience of the formation of the perception of product quality, which, in turn, constructs a strong knowledge base, helping to resist the influence of information overload.

### 5.2. Theoretical contributions

Previous studies have analyzed the impact of information overload on user behavior from the perspective of HSM, but have not explained exactly what systemic and heuristic factors are affected by information overload [15]. This study first refines the factors affecting users’ purchase intention in live commerce from the standpoint of HSM (expanding its connotation), such as perceived product quality and fit, and streamer influence and professionalism. Second, this study introduces the information overload aspect, and analyzes its impact on specific factors in users’ system information processing and the information processing process, and confirms the role of information overload marketing in live commerce, thereby not only enriching the literature on live commerce but also expanding the antecedents of HSM. Finally, the impact of information overload on users’ willingness to consume has been controversial [13], often with different effects in different contexts. This study, on the other hand, confirms the effect of information overload on users’ purchase intentions in a live commerce environment, and contributes to the development of information overload theory in marketing.

### 5.3. Practical contributions

This study also offers practical guidance in the following ways. First, live business practitioners should strictly control the quality. Consumers’ perception of product quality is based on the accumulation of long-term shopping experiences, and it is difficult for any marketing to change consumers’ attitudes toward poor-quality products. This requires the live broadcast to be well-prepared for various activities, convey a detailed understanding of the products it promotes, and be able to recommend products and diversify marketing. In particular, we screen products strictly in the primary selection, control their quality, and ensure that the quality of products recommended to consumers is consistent with their actual quality. This is to improve the quality of products perceived by users, prevent consumers from purchasing inferior products, reduce after-sales problems, continue to win through word-of-mouth, and accumulate users.

Second, streamers should constantly improve their influence. The length of a live streaming, which is mostly five to six hours, is a test of a person’s psychology and body; streamers need to have the perseverance to grasp the rhythm of a live broadcast. Streamers must also be patient, and insist on maintaining high passion and emotion in front of the camera for a long time, while maintaining their own personal style and not blindly imitating others. The longer the live broadcast, the more followers it has, and the stronger its influence will be. Third, streamers should pay attention to the packaging outside the live channel, such as shooting beautiful short videos to package themselves; this not only attracts new fans but also allows existing fans to understand the streamer’s information even when they are not watching live. Streamers can also occasionally organize offline meetups for fans and perform thanksgiving feedback activities to increase their popularity and attract new fans. Additionally, the influence cannot be separated from the rules of the platform. We should understand the promotion rules of the platform; otherwise, even if the streamer is excellent and markets a good product, it is not easy to represent or display it.

Finally, streamers can cleverly use the influence of "information overload." In a limited period of time, they can provide consumers with a large amount of information in various forms, such as repeatedly emphasizing the strong points of the product, constantly executing limited-time rush activities, holding positive emotional interaction with consumers, and creating a hot live-room environment, etc.; these efforts touch the pain points of consumers directly, so that they cannot think carefully about the core features of the product, and always follow the guidance of the streamer.

### 5.4. Limitations and future directions

There are also some shortcomings in this study, which are expected to be addressed in future studies. First, in live commerce, users’ attitudes toward products are influenced not only by the marketing approach of the streamer, but also by other consumers, e.g., by the herding effect. The present study did not consider such influences, especially from other consumers of live commerce, and encourages future studies to introduce HSM for validation. Second, HSM theory also suggests that there is an interplay between system processing factors and heuristic information processing factors, which is also not analyzed in this study, and future studies are encouraged to analyze this aspect. Finally, this study did not consider comparisons among multiple product types, and it is hoped that future studies will conduct further analysis along those lines.

## Supporting information

S1 Appendix Measurement items. (DOCX)

S1 Data (ZIP)
