# Supplementary material for: Examining the influence of information overload on consumers’ purchase in live streaming: A heuristic-systematic model perspective
Source: PLoS One. 2023 Aug 4;18(8):e0284466. doi: 10.1371/journal.pone.0284466 (PMC10403099; doi:10.1371/journal.pone.0284466)
Supplement: S1 Appendix — (DOCX) [file pone.0284466.s001.docx]

## Measurement items

Appendix A. Measurement items

| **Factors** | **Serial Num.** | **Item** | **Reference** |
| --- | --- | --- | --- |
| Perceived Product Quality | PPQ1 | I feel that the streamer recommended items have good performance. | Piri and Lotfizadeh [58] |
|  | PPQ2 | I felt that the overall quality of the items recommended by the streamer was very high. |  |
|  | PPQ3 | I feel that the quality standards followed by the merchandise recommended by the streamer are high. |  |
| Perceived Product Fit | PPF1 | I think the products recommended by the streamer fit my taste. | Lu and Chen [60] |
|  | PPF2 | I think the products recommended by the streamer will fit my preferences. |  |
|  | PPF3 | The streamer's recommendation does not reduce my concern about the suitability of the product (reverse). |  |
| Streamer Influence | SIF1 | I often watch the streamer who has more fans. | Ryu and Han [40] |
|  | SIF2 | The streamers I watch regularly have a great influence. |  |
|  | SIF3 | The streamers I watch regularly are highly visible. |  |
| Streamer Expertise | STE1 | The streamers I often watch prepare more professional content than others. | Ryu and Han [40] |
|  | STE2 | I often watch streamers who are very knowledgeable about the goods for sale. |  |
|  | STE3 | The streamers I often watch have very specialized knowledge in the field of live business. |  |
| Purchase Intention | PIT1 | Through the live broadcast, I will consider purchasing the recommended products. | Chang, Lu and Lin [31] |
|  | PIT2 | The likelihood of my purchasing products recommended through live streaming is high. |  |
|  | PIT3 | Through the live broadcast, I was interested in purchasing the recommended products. |  |
| Information Overload | IFO1 | The streamer gave me too much information and I couldn't focus on the product. | Cao and Sun [25]  Farooq, Laato, Islam and Isoaho [28] |
|  | IFO2 | The streamer sent me too much information, and I lacked the ability to use it to evaluate the product. |  |
|  | IFO3 | The streamer sent me so much information that I had difficulty using it to evaluate the product in a comprehensive way. |  |
